# Development and Validation of Medical Students’ Professionalism Assessment Scale (MSPAS) In OSCE: Simulated Patients’ rating scale

**DOI:** 10.15694/mep.2019.000094.1

**Published:** 2019-04-22

**Authors:** Mila Nu Nu Htay, Saurabh Shrivastava, Soumendra Sahoo

**Affiliations:** 1 Melaka Manipal Medical College

**Keywords:** professionalism, assessment, simulated patient, medical students, scale, validated

## Abstract

This article was migrated. The article was marked as recommended.

Background

To provide the reliable and immediate feedback on the medical students’ professionalism during the OSCE, we developed a modified Medical Students’ professionalism assessment scale (MSPAS) which is relevant in the history taking and physical examination OSCE stations.

Methods

We developed two sets of questionnaires, Medical Students’ Professionalism Assessment Scale- History taking (MSPAS-H) and Medical Students’ Professionalism Assessment Scale- Physical Examination (MSPAS-PE).The Cronbach’s alpha correlation coefficient was calculated to determine the internal consistency and exploratory factor analysis was carried out.

Results

The Content validity index was calculated by using the expert’s rating on relevance and all the 12 items in the MSPAS had above 0.85 of CVI. Reverse scoring was required for item No 4 (MSPAS-H) and item No 5 (MSPAS-PE). The Cronbach’s alpha was calculated for reliability and found to have 0.87 for MSPAS-H and 0.92 for MSPAS-PE.

Discussion

All the 6 items in the history taking and 6 items in the physical examination were loaded in each scale with the factor loading of 0.5 and above for all the items. This MSPAS is short and simple scale, it can be completed in a short time period if the simulated patients are provided the proper training on this assessment.

## Introduction

### 1. Introduction

The society expects doctors who are not only clinically skillful but behave in an ethical and professional manner. The term Professionalism encompasses: integrity, honesty, compassion, awareness of recent advances, communicate effectively and respect for patients. The onus lies with the medical schools to inculcate professional behavior and teach ethics to the medical students and also develop an assessment process to assess the professional development.

Physical examination techniques, professionalism, attitudes for effectively interacting with patients and communication skills are best assessed during actual performance. Examinations that assess skills during actual performance are known as *competency-based* or *performance-based* assessments. Among the various types of competency based tests, OSCEs are most widely used because they are valid, reliable, and fair. OSCE are Objective in that content and scoring procedures are standardized. Each examination station is designed to focus on an area of clinical competence. A standardized scoring tool is used to record what you do or do not do well. They are structured meaning every OSCE candidate experiences the same problem, and is asked to perform the same task, within the same timeframe. It is Clinical in that the tasks in each OSCE station represent real-life clinical situations. And assess the ability to apply clinical knowledge and skills and finally Examinationmeaning an OSCE enables a reliable assessment of a candidate’s competence ( MCC, 2019).

In OSCE the medical students interact with a series of simulated patients (SP) in stations that may involve history-taking, physical examination, counselling or patient management. SPs are individuals trained to perform the role of a patient realistically and consistently. An advantage of simulated patients over real patients is that of allowing different students to be presented with a similar challenge, thereby reducing an important source of variability .Other advantages include their reliable ,availability and adaptability, which enables there production of a wide range of clinical phenomena tailored to the student’s level of skill ( Wallace, Rao and Haslam, 2002).

Assessment is fundamental part in medical education and it should include not only the attainable knowledge and skills but also the professionalism and communication ( Klemenc-Ketis and Vrecko, 2014). Several studies have investigated the professionalism among the medical students and trainees ( Klemenc-Ketis and Vrecko, 2014; Gale-Grant, Gatter and Abel, 2013; Tsai *et al.*, 2007). In these studies, the participants recognized professionalism as confidentiality, good knowledge and skills, respect to the patients and accountability ( Gale-Grant, Gatter and Abel, 2013; Tsai *et al.*, 2007; Gillespie *et al.*, 2009). The professionalism is crucial in both doctor-patient communication and inter-professional communication. A systemic review of instruments to assess professionalism mentioned that scales have been developed for the undergraduate medical students and practicing health care professionals by using direct observation assessment, self-administered questionnaires, multi-source feedback, and simulation ( Li *et al.*, 2017).

The simulated patients are unique as they can be trained to provide the reliable and immediate feedback on the medical students’ professionalism during the OSCE. However, one of the limitations is the time constraints for the simulated patients to provide the immediate assessment for the students after they answered at the history taking station or they are being examined at the physical examination station. Therefore, our study team planned to develop a short and simple scale to assess the professionalism and communication skills from the perspective of the simulated patients. Thorough literature search had been conducted to develop the items to be included in the scale. Hurst Y, et al (2004) had developed the questionnaire to assess the vocational dental practitioner’s interpersonal skills ( Hurst, Prescott-Clements and Rennie, 2004). Some of the items were considered to be relevant in the OSCE setting and therefore, we have modified and proceeded to develop a scale which is relevant in the history taking and physical examination OSCE stations.

To the best of our knowledge, there is no study of using medical students’ professionalism assessment scale during OSCE in Malaysia. In the study institution, the medical students begins their clinical posting in semester 6 (third year) and the final phase II stage II (PII SII) examination is conducted at the end of semester 9 (fifth year). Upon completion of the semester 9, the second half of the final year (semester 10) will be trained in the clinical settings only, which is known as the shadow-housemanship training. Therefore, we have decided to assess the medical students’ professionalism with the simulated patients’ rating during the PII SII OSCE examination before the students enter to the clinical training in semester 10. Our study aimed to develop the medical students’ professionalism assessment scale in OSCE rated by simulated patients.

## Methods

### 2. Method

#### 2.1. Development of Medical Students’ Professionalism Assessment Scale

In this study, the items for the history taking and physical examination stations were developed by using two different approaches. The first approach was literature search, and adopting some items from the previous studies on the professionalism assessment ( N.R. Aravamudhan and Krishnaveni, 2015). As a second approach, the qualitative focus group discussion with nine members of the OSCE committee was conducted to identify and decide the critical items to assess the students’ professionalism. Finally, the total number of 6 items were developed for each of the history taking and physical examination stations. Each item was given the scoring from 0 (strongly disagree) to 10 (strongly agree).

#### 2.2. Content validity

The validation of the MSPAS was initiated by collecting the opinions from the expert judges to establish the content validity. Content validity is the initial and important step in developing the scale. In our study, the content validation was established by requesting the experts’ opinion and analyzing their ratings ( N.R. Aravamudhan and Krishnaveni, 2015).

##### 2.2.1. Expert participation

The experts were selected from the medical educationists, community medicine and primary healthcare specialist and the clinicians. They were requested to provide their opinion on each of the items of MSPAS. The study information sheet, the guiding for the expert opinion form and the opinion rating sheets were sent to the six experts via email. The rating sheet included three parts of assessment for each items, (1) ‘ *Relevant rating*’ (scale 1- 4), (2) ‘ *Is the item well written?*’ and (3) ‘ *Is the item essential to the domain/ category*?’ The confidentiality was maintained and the experts were provided two-week time to go through items and to provide the ratings.

##### 2.2.2. Analysis of experts’ rating

The ratings of the experts were recorded in the excel file and provided the code number for each expert in order to ascertain the anonymous ratings. According to the N.R. Aravamudhan, et al (2015), the content validation could establish by analyzing both descriptive and quantitative methods ( N.R. Aravamudhan and Krishnaveni, 2015). For the descriptive analysis, the item ambiguity, medium, percentage agreement were calculated. For the quantitative analysis, the content validity index, content validity ratio, content validity coefficient were used to establish the validity for each items ( N.R. Aravamudhan and Krishnaveni, 2015).

#### Medium

The experts were requested to provided rating on the relevance of the item as follow; (1) Not relevant, (2) Not important, (3) Relevant and (4) Very important.

#### Item ambiguity score

The item ambiguity score for each item was calculated by using the formula of

R _k_ = X _KjH_ - X _KjL_

in which “X _KjH_ is the highest ranting and X _KjL_ is lowest rating”. The R _k_ of 3 and above were considered as the highly ambiguous items ( N.R. Aravamudhan and Krishnaveni, 2015).

#### Percentage agreement

The percentage agreement was calculated based on the expert’s opinion on the essentiality of the item in the score. The percentage agreement is “(the number of experts rated essential/ total number of experts) x 100 ( N.R. Aravamudhan and Krishnaveni, 2015). The items with percentage agreement of 80% and above were decided to retain in the scale ( N.R. Aravamudhan and Krishnaveni, 2015).

#### Content validity index (CVI)

The content validity index was calculated by using the expert’s rating on relevance, in which the scores were provided from 1 to 4. If the expert’s rating is 3 or 4, that item was considered to be relevant and if the rating is 1 or 2, it was considered as not relevant for this scale. The CVI was calculated as follow;

CVI = (No of experts rated 3 or 4/ Total no of experts) ( N.R. Aravamudhan and Krishnaveni, 2015). The CVI of 85% or 0.85 and above were decided to be retained in the scale ( Lynn, 1986).

#### Content validity ratio (CVR)

The content validity ratio was calculated based on the experts’ rating on essential item.

CVR = (n _e_- N/2) / N/2

In which n _e_ is the number of experts rated as essential and N is the total number of experts ( N.R. Aravamudhan and Krishnaveni, 2015). The CVR value of 0.99 was set as the acceptable value as there were 6 experts in our study ( Lawshe, 1975).

Content validity coefficient (VIk)

As the final step of content validation, VIk was calculated by using the following formulae;

S _j_ = r _j_ - L _o_

  L _o_ = the lowest validity category

  r _j_ = the expert’s (J’s) rating

VI _k_ = S/ [j (c-1)]

  S = the sum of S _j_

  C = number of rating categories (1 to 4 in this study)

  J = the number of experts ( Aiken, 1985; N.R. Aravamudhan and Krishnaveni, 2015).

In our study, we collected six expert’s opinions with 4 ratings, so that VIk of 0.78 was decided as acceptable ( Aiken, 1985).

#### 2.3. Construct validity

##### 2.3.1. Data collection

The MSPAS-H and MSPAS-PE in OSCE stations included 6 items in each rating scale. The data were collected during the Phase II Stage II OSCE examination in a private medical institution in Malaysia. The simulated patients were used during the OSCE and they were provided the training and instruction on how to provide the professionalism rating to the medical students.

Regarding the history taking (MSPAS-H), four simulated patients at the family medicine OSCE stations provided the ratings. For the physical examination (MSPAS-PE), eight simulated patients at the ophthalmology physical examination OSCE stations provided the assessment for the medical students. The rating sheets were provided code numbers and data were entered and saved in the Microsoft excel file.

##### 2.3.2. Reliability analysis

The Cronbach’s alpha correlation coefficient was calculated to determine the internal consistency of each of the history taking and physical examination professional assessment scales. Reverse scoring was required for item No 4 (MSPAS-H) and item No 5 (MSPAS-PE).

##### 2.3.3. Factor analysis

The exploratory factor analysis was carried out to examine the domains and the factor loading of each items to their respective domains. Principle component extraction method and Promax rotation was used during the analysis by using PASW (version 18).

This study was approved by institutional research & ethics committee.

## Results/Analysis

### 3. Results

The total number of 117 students for history taking station and 127 students for physical examination station were assessed their professionalism by the simulated patients.

#### 3.1. Content validity

##### 3.1.1 Descriptive analysis

The medium scores of ratings for the items ranging from 3 to 4. Meanwhile, the item ambiguity score were ranging between 0- 1. Regarding the percentage agreement, 10 out of 12 items had the percentage agreement of 83 and above. The remaining 2 items had 67% of agreement. However, these two items were still retained to compare with the other analysis results to make the decision of deletion ( Table 1).

##### 3.1.2. Quantitative analysis

All the 12 items in the MSPAS had above 0.85 of Content validity index (CVI). Content validity ratio (CVR) of 3 items from the history taking and 3 from the physical examination had less than 0.99. In our study, the Content validity coefficient (VI _k)_ of the items were ranging from 0.78 to 1 ( Table 1).

The decision to retain or delete the items in the scale was decided based on the number of methods that the individual items was accepted. Among the 6 method of content validation, the item accepted in at least 4 methods (66.67% acceptance) was decided to retain in the scale ( N.R. Aravamudhan and Krishnaveni, 2015) ( Table 1).

**Table 1.** Content validation of the items in Medical Students’ Professionalism Assessment Scale

Item No	Medium	Item ambiguity score	Percentage agreement (%)	CVI	CVR	VI _k_	Decision
**History Taking**							
1	4	0	100	1	1	1	Retain
2	4	1	100	1	1	0.89	Retain
3	3.5	1	83	1	0.67	0.83	Retain
4	3.5	1	83	1	0.67	0.83	Retain
5	4	0	100	1	1	1	Retain
6	4	1	83	1	0.67	0.89	Retain
**Physical Examination**							
1	4	0	100	1	1	1	Retain
2	3	1	67	1	0.33	0.78	Retain
3	4	1	100	1	1	0.89	Retain
4	4	0	100	1	1	1	Retain
5	3.5	1	67	1	0.33	0.83	Retain
6	4	1	83	1	0.67	0.94	Retain

#### 3.2. Construct validity

##### 3.2.1. Reliability analysis

In this study, the Cronbach’s alpha value of MSPAS-H is 0.87 and MSPAS-PE is 0.92 ( Table 2).

##### 3.2.2. Factor analysis

The Kaiser Meyer Olkin (KMO) of MSPAS-H is 0.8 and MSPAS-PE is 0.83. In the exploratory factor analysis, the percentage of variance for MSPAS-H is 62% and for MSPAS-PE is 72%. The factor loading for MSPAS-H is ranging from 0.5-0.94, meanwhile, for MSPAS-PE is ranging from 0.65-0.93 ( Table 3).

**Table 2.** Reliability test of Medical Students’ Professionalism Assessment Scale

Item No		Corrected-Item Total Correlation	Cronbach’s Alpha if Item Deleted	Number of items
**History Taking**				
1	The student addressed me respectfully/ politely.	0.56	0.86	6
2	The student’s questions were clear to me.	0.87	0.81	
3	The student asked thoughtful questions.	0.83	0.82	
4	The student interrupted me while I was talking.	0.38	0.89	
5	The student explained what I need to know about my problems.	0.83	0.82	
6	I would you like to be interviewed by this student again.	0.58	0.87	
Overall			**0.87**	
**Physical Examination**				
1	The student addressed me respectfully/ politely.	0.89	0.89	6
2	The student’s questions were clear to me.	0.85	0.90	
3	The student gave clear instructions before examining me.	0.85	0.90	
4	The student was gentle in doing physical examination on me.	0.88	0.89	
5	The student caused excessive/ unnecessary pain during examining me.	0.54	0.94	
6	I would you like to be examined by this student again.	0.66	0.92	
Overall			**0.92**	

**Table 3.** Factor loading for each items in Medical Students’ Professionalism Assessment Scale

Item	Factor Loading
**MSPAS-H**	
1	0.67
2	0.94
3	0.91
4	0.50
5	0.91
6	0.74
**MSPAS-PE**	
1	0.93
2	0.91
3	0.91
4	0.92
5	0.65
6	0.75

## Discussion

The professionalism is essential to develop during the medical students’ life, and continue to practice throughout the career. In the modern world much stress is given to teach these competencies viz; professional behavior and ethics to the medical students and also assess the professional development. Simulated patients provide feedback to undergraduate medical students on their performance in a variety of domains, such as interviewing skills or physical examination skills, and in a variety of formats, such as verbally or with the use of written checklists ( Bokken *et al.*, 2009) & the checklist component was reflected in our study as well.

In our study the content validation is established to determine whether the items in the scales are relevant to for the assessment of professionalism among the medical students. The developed scale by us was validated by descriptive and quantitative analysis similar to as described in literature (N.R.Aravamudhan and Krishnaveni, 2015).

We analyzed the internal consistency to investigates how the items are correlated to each other within the instrument as described in literature ( Parsian and AM, 2009). The Cronbach’s alpha was calculated to assess the internal consistency and value of >0.7 is considered as an acceptable internal consistency among the items in the instrument described in literature ( Tavakol and Dennick, 2011) & our instrument found to have Cronbach’s alpha of 0.87 for MSPAS-H & 0.92 for MSPAS-PE, which indicated that the items in MSPAS were considered to have good internal consistency levels.

The construct validation was demonstrated by using the exploratory factor analysis, which was intended to assess the theoretical construct of the MSPAS. It is an important method to development the scale, refine the items and evaluate the measurement instrument or scale ( Williams, Brown and Onsman, 2010). The exploratory factor analysis method is widely used in developing the instrument in education, social sciences, psychology, etc ( Taherdoost, Sahibuddin and Jalaliyoon, 2014). During the EFA, the (KMO) correlation was calculated to assess the sampling adequacy for this study. The KMO correlation of “0.5 is considered acceptable, 0.5 - 0.7 is mediocre, 0.7 - 0.8 is good, 0.8-0.9 is great and >0.9 is superb” ( Parsian and AM, 2009). In this study, KMO was 0.8 and 0.83 for MSPAS-H and MSPAS-PE respectively. Therefore, the sample size of this study was considered as adequate to proceed to the factor analysis.

In the exploratory analysis, the 6 items in the history taking and 6 items in the physical examination were loaded in each scale with the factor loading of 0.5 and above for all the items. This finding demonstrated that the relationship of each variable to the underlying factor is acceptable.

This MSPAS is short and simple scale, it can be completed in a short time period if the simulated patients are provided the proper training on this assessment. In this pilot study, the MSPAS had found to have good and reliable psychometric value. The strength of our study was our instrument which was used during two different type of clinical competency testing at OSCE (Communication skill during history taking & during physical examination) & also the instrument was used at real examination setting. However the limitation was smaller sample size & use of only English version of scale. In order to reflect the local setting, it would be better if the scale is translated and validated to local language of the simulated patients. Further studies with the larger student sample is recommended to proceed for the confirmatory factor analysis to be undertaken and for the generalizability of the scale.

## Conclusion

This study reveals that the MSPAS is a valid and reliable scale to assess the medical student’s professionalism during the OSCE.

## Take Home Messages

• There should be assessment for the professionalism of medical students
• Professionalism assessment during OSCE setting is possible
• The Simulated Patient testing professionalism is ideal way during OSCE
• A validated MSPAS can be more authentic way of testing professionalism

## Notes On Contributors

Mila Nu Nu Htay:

Dr. Mila Nu Nu Htay is working as an Assistant Professor in the Department of Community Medicine. She completed her M.B., B.S (MDY), DTM&H (London) and MTID (UK) and currently pursuing Ph.D. (Public Health).

Saurabh Shrivastava:

Dr Saurabh Shrivastava is working as Associate Professor in the Department of Ophthalmology. She did her MS in Ophthalmology from Delhi University .She has 17 years of teaching experience in post graduate medical college and has 18 research papers to her credit and has guided 3 post graduate research projects.

Soumendra Sahoo:

Professor Soumendra Sahoo with 25 years of teaching experience is currently Head of Ophthalmology & Chair Medical Unit at MMMC. He obtained his PhD in Nanomedicine from Utkal University, India. He had successfully completed FAIMER Fellowship on Medical Education in 2014 & is an International Faculty member of CMCL-FAIMER institute.

## Declarations

The author has declared that there are no conflicts of interest.

## Ethics Statement

The study protocol was submitted to the Research Ethics Committee, Faculty Of Medicine, Melaka-Manipal Medical College. The Ethics Committee reviewed the protocol and granted the approval to conduct the study (Ref: MMMC/FOM/Research Ethics Committee -12/2018).

## External Funding

This article has not had any External Funding
