# Supplementary material for: Development and Validation of Medical Students’ Professionalism Assessment Scale (MSPAS) In OSCE: Simulated Patients’ rating scale
Source: MedEdPublish (2016). 2019 Apr 22;8:94. [Version 1] doi: 10.15694/mep.2019.000094.1 (PMC10712613; doi:10.15694/mep.2019.000094.1)
Supplement: Peer review history

# Reviewer report: Reviewer response for version 1

**DOI:** 10.21956/mep.19835.r29904

**Published:** 2019-04-25

**Authors:** BALAJI ARUMUGAM

**Affiliations:** 1 TAGORE MEDICAL COLLEGE AND HOSPITAL

This review has been migrated. The reviewer awarded 4 stars out of 5 Thanks for the invite. I appreciate the work of the authors in developing the professionalism assessment scale which is the thoughtful idea and need of the hour to quantify the learning experience of the learners. The article was also well written and scientifically, statistically proven concepts were utilized to assess the scale measurements (Content validity, reliability testing and calculation of Cronbach’s alpha which was more than 0.8 in all the factors, with factor analysis). The scale is also short and crisp including all the dimensions of the professionalism.

Reviewer Expertise:

NA

No decision status is available

# Reviewer report: Reviewer response for version 1

**DOI:** 10.21956/mep.19835.r29901

**Published:** 2019-04-25

**Authors:** Balakrishnan Nair

**Affiliations:** 1 Centre for Medical Professional Development and University of Newcastle

This review has been migrated. The reviewer awarded 4 stars out of 5 Assessing professionalism is important in medical education at all levels. However this is often not done. So this study to develop a tool and evaluate professionalism assessment is worthwhile. The methodology and sample size are good; the analysis is very appropriate and accurate. Some aspects of professionalism like teamwork and entrustable professional activities are challenging to assess in OSCE setting The next level could patients evaluating students , even though there are logistical and sample size issues. Well done

Reviewer Expertise:

NA

No decision status is available

# Reviewer report: Reviewer response for version 1

**DOI:** 10.21956/mep.19835.r29905

**Published:** 2019-04-23

**Authors:** P Ravi Shankar

**Affiliations:** 1 American International Medical University

This review has been migrated. The reviewer awarded 4 stars out of 5 This is an interesting study regarding the development and validation of the Medical Students’ Professionalism Assessment Scale (MSPAS). The scale seems short and reliable for use during OSCEs. The scale seems simple enough to be used by simulated patients. The process of development of the instrument has been well described. The study is mostly well written though corrections to language and grammar may be required in a few places. The authors have not described their scale in the main paper. They have included the statements constituting the scale only in table 2. This may make it difficult for a reader to understand the instrument. Statement 6 in both the history and physical examination scale may need language corrections. The scale seems short and simple enough for use. However, it may require to be tested among a bigger and wider population of students. All educators will find this article of interest.

Reviewer Expertise:

NA

No decision status is available

# Reviewer report: Reviewer response for version 1

**DOI:** 10.21956/mep.19835.r29903

**Published:** 2019-04-23

**Authors:** Avinash Supe

**Affiliations:** 1 Seth G S Medical College

This review has been migrated. The reviewer awarded 4 stars out of 5 Thank you for opportunity for review This is excellent process by which professionalism can be assessed for history as well as clinical examination. Authors have used valid psychometric process . We appreciate their effort. It must be reliably used while implementing

Reviewer Expertise:

NA

No decision status is available

# Reviewer report: Reviewer response for version 1

**DOI:** 10.21956/mep.19835.r29902

**Published:** 2019-04-22

**Authors:** Lee HangFu

**Affiliations:** 1 Windsor University School of Medicine

This review has been migrated. The reviewer awarded 4 stars out of 5 Thank you for the opportunity to review this paper. The use of scientific analysis gives credibility to this professionalism assessment. The psychometric analysis approach lends validity to medical education assessments. The items used in the assessments are generic, therefore, individual Medical School must identify and apply the items reliably to the professionalism Learning Objectives specific to individual medical School Curriculum.

Reviewer Expertise:

NA

No decision status is available
